# General Medical Council of Great Britain

**Published:** 1898-10

**Authors:** 


### GENERAL MEDICAL COUNCIL OF GREAT BRITAIN.

This very important body has had added to its membership Mr. Charles S. Tomes. This has only been accomplished after a long struggle, in which our colaborers on the other side had to contend with medical prejudice and custom. That they finally conquered is a positive credit, but that they succeeded in securing as member Mr. Tomes is worthy of special congratulation. He is certainly the right man for the position.

It is quite evident, from the abstracts given upon another page, that this body sadly needed missionary work; at all events, the benighted Sir C. Nixon needs enlightenment and conversion. It seems passing strange that any one could be found willing to give utterance to the following: "Why a dental student should have a knowledge of the materia medica I really cannot understand." Sir Nixon has probably passed the age when revelations come to men, but it would not be unworthy even his high position to seek wisdom by a closer study of dentistry and its relations. In American dental schools, one and frequently two sessions are devoted to the study of drugs, in order that the therapeutics of dentistry may rest upon a solid foundation. This is equally as well understood in England, and why the council should quibble over it seems passing strange.

In reading the proceedings, not only of this council, but of all gatherings in Great Britain, one is impressed with the dignity which accompanies the proceedings. This might be copied with benefit in this country.

